# Impacts of Climatic Variability on *Vibrio parahaemolyticus* Outbreaks in Taiwan

**DOI:** 10.3390/ijerph13020188

**Published:** 2016-02-03

**Authors:** Hsin-I Hsiao, Man-Ser Jan, Hui-Ju Chi

**Affiliations:** 1 Department of Food Science, National Taiwan Ocean University, 2 Beining Road, Keelung 202, Taiwan; hi.hsiao@ntou.edu.tw (H.-I.H.); bluesky3773@hotmail.com (H.-J.C.); 2 Institute of Applied Economics, National Taiwan Ocean University, 2 Beining Road, Keelung 202, Taiwan

**Keywords:** *Vibrio parahaemolyticus*, food poisoning, outbreak, climate, variability, Taiwan

## Abstract

This study aimed to investigate and quantify the relationship between climate variation and incidence of *Vibrio parahaemolyticus* in Taiwan. Specifically, seasonal autoregressive integrated moving average (ARIMA) models (including autoregression, seasonality, and a lag-time effect) were employed to predict the role of climatic factors (including temperature, rainfall, relative humidity, ocean temperature and ocean salinity) on the incidence of *V. parahaemolyticus* in Taiwan between 2000 and 2011. The results indicated that average temperature (+), ocean temperature (+), ocean salinity of 6 months ago (+), maximum daily rainfall (current (−) and one month ago (−)), and average relative humidity (current and 9 months ago (−)) had significant impacts on the incidence of *V. parahaemolyticus*. Our findings offer a novel view of the quantitative relationship between climate change and food poisoning by *V. parahaemolyticus* in Taiwan. An early warning system based on climate change information for the disease control management is required in future.

## 1. Introduction

Fish and seafood are central to the diet in Taiwan. The average per person fish consumption in Taiwan was 36.5 kg/yr in 2012, which was 2.5 times higher than the world average of 14.5 kg/yr. In Taiwan, the premier food poisoning threat over the most recent ten years has been *Vibrio parahaemolyticus*, and seafood is the major food type causing food poisoning (Table 1) [1,2]. *V. parahaemolyticus* is naturally present in the marine and estuarine environments of tropical and temperate areas. It is naturally of particular importance in the countries where seafood consumption is high [3,4,5]. *V. parahaemolyticus* is a Gram-negative, halophilic bacterium. There are three major manifestations of *Vibrio* infection: gastroenteritis, wound infection, and primary septicemia. Fatality rates were 1% for gastroenteritis, but as high as 5% for wound infections and 44% for septic disease [6]. Although the microbiological aspects of seafood safety have been studied intensively for many decades, there is still a considerable burden of food‑borne illness, even in industrialized countries [7,8,9,10].

Climate change or variability is the greatest environmental challenge the world faces today [11,12,13,14]. Climate change includes changes in temperature and precipitation patterns, increased frequency and intensity of extreme weather events, ocean warming and acidification, and changes in the transport pathways of complex contaminants [15]. A substantial body of influential research indicates that climate change relates to food safety because it affects microbial ecology and growth, plant and animal physiology and host susceptibility [16,17,18]. These effects, in turn, could affect human health [11,19,20,21,22,23,24,25].

Quantitative data have indicated that climate variation is related with infectious diseases caused by pathogenic organisms, such as *Salmonella* [22,26], pathogenic *Escherichia coli* [23], and *Campylobacter* [27]. Although growing numbers of researchers have considered the influences of climate change on human health caused by pathogenic organisms, very little attention has been paid specifically to *V. parahamolyticus*, and from the perspective of Taiwan [28]. This paper therefore serves to address this under-researched area. We aim to quantify the relationship between climate variability and *V. parahaemolyticus* incidence in Taiwan in order to provide a mechanism with which to establish an early warning system in the future.

## 2. Materials and Methods

### 2.1. Materials/Data

Monthly data for 2000–2011 of *V. parahaemolyticus* outbreaks (outbreak) was obtained from the Food and Drug Administration, Department of Health, Taiwan due to data access limitations. The climate data was obtained from the Central Weather Bureau, Taiwan, and were aggregated from all recording stations. They were maximum, average and minimum temperature (maxtemp, avgtemp, mintemp), maximum, average, and minimum relative humidity (maxrh, avgrh, minrh), as well as average and maximum daily rainfall (avgrf, maxrfd). Data for oceanic temperature and salinity (octemp, ocsant) were also obtained from Ministry of Science and Technology, Taiwan. A total of 144 monthly data values were obtained. The study area range is at latitude 20°00’North to 29°58’North and longitude 115°00’East to 124°99’East. Regional differences were not considered in this study because Taiwan is a small island [21].

### 2.2. Methods/Analysis

In order to accommodate the autocorrelation and seasonality in the time course of *V. parahaemolyticus* outbreak cases, we employed a Box & Jenkins [29] seasonal autoregressive integrated moving average model (ARIMA). This model captured variation, autocorrelation, long-term trends, and allowed the examination of the independent impacts of the covariates such as the climatic variables. This model has been widely used to examine the seasonality in the time course of other pathogens, such as *Salmonella* [22,26], pathogenic *E. coli* [23], and *Leptospira interrogans* [30].

The seasonal ARIMA model incorporates both non-seasonal and seasonal factors in a multiplicative model. One shorthand notation for the model was ARIMA (p,d,q)×(P,D,Q)_S_. The model could be written more formally as:

(1) $$(1-B^{S}{)}^{D}\Phi (B^{S}){\left(1-B\right)}^{d}\phi (B)\;y_{t}=\Theta (B^{S})\;\theta (B)\;\varepsilon_{t}$$

where *y_t_* was the outbreak variable, *ε_t_* was a white noise process, *B* was lag (or back) operator; non-seasonal components AR(p): *ϕ*(*B*) = 1 − *ϕ*_1_(*B*) − *ϕ*_2_(*B*)^2^ −…−*ϕ*_p_(*B*)*^p^*, MA(q): *θ*(*B*) = 1 + *θ*_1_(*B*) + *θ*_2_(*B*)^2^ +…+ *θ*_q_(*B*)*^q^*; seasonal components AR(P): φ(*B^S^*) = 1 − φ_1_(*B^S^*) − φ_2_(*B^2S^*)^2^ −…−φ_p_(*B^PS^*), MA(Q): Θ(*B^S^*) = 1 + Θ_1_(*B^S^*) + Θ_2_(*B^2S^*)^2^ +…+ Θ_Q_(*B^QS^*), and *d* was the degree of non‑seasonal differentiation, *D* was the degree of seasonal differentiation, and *S* was the length of the seasonal cycle. On the left side of Equation (1) the seasonal and non-seasonal AR components multiplied each other, and on the right side of Equation (1) the seasonal and non-seasonal MA components multiplied each other. The seasonal AR components captured the seasonal pattern of the dependent variable, and non-seasonal AR components captured the lag-time structure of the dependent variable.

The outbreak variable was first tested for seasonal variation and stationarity by the Augmented Dickey-Fuller (ADF) unit root test. The orders of the ARIMA(p,d,q) × (P,D,Q)_S_ model were then determined by examining the autocorrelation function (acf) and the partial autocorrelation function (pacf). A detailed explanation of ARIMA procedures was provided in Box and Jenkins [29]. The climate variables were included in the model by calculating the cross-correlations function (ccf) to examine the correlation between the climatic variables and the *V. parahaemolyticus* outbreaks. All ARIMA modelling and the corresponding statistical tests were performed in the SAS/ETS 9.3 statistical software.

## 3. Results

To avoid the collinearity problem of the independent variables, we calculated the Pearson correlation coefficients between all pairs of climate variables (Table 2). The temperature variables, maxtemp, avgtemp, and mintemp, were highly correlated (all *r* > 0.99) with each other, and variable avgtemp had the highest correlation with outbreaks. We therefore selected avgtemp as the temperature variable. For the same reason, maxrfd was selected as the rainfall variable and avgrh that for relative humidity. *Vibrio* incidence was also significantly and positively correlated with average temperature (avgtemp) (*r* = 0.2675), maximum daily rainfall (maxrfd) (*r* = 0.1402), and ocean temperature (octemp) (*r* = 0.1938). Average relative humidity (avgrh) and ocean salinity (ocsant) were not clearly correlated with *Vibrio* outbreaks.

We summarized the descriptive statistics of outbreaks and climate variables in Table 3. The average values, maximum values and minimum values of outbreak variable and climatic variables were calculated by month from 2000 to 2011. In total, 3870 *V. parahaemolyticus* outbreaks occurred between 2000 and 2011 in Taiwan. On average, 26.9 cases each month occurred shown in the last column of Table 3. Over 80% of incidences occurred between May and September which were the warm, humid and rainy months in Taiwan. The average temperature (avgtemp) during these months was about 3.94 °C higher than the annual average temperature, the average maximum daily rainfall was 37.36 mm more than the annual average (69.4 mm), and the average ocean temperature was 0.38 °C higher than the annual average (20 °C). In contrast, the ocean salinity during this period was 0.18 Practical Salinity Unit (psu) lower than the average (34.1 psu). Outbreaks and all these climatic variables showed annual seasonality.

**Figure 1:**
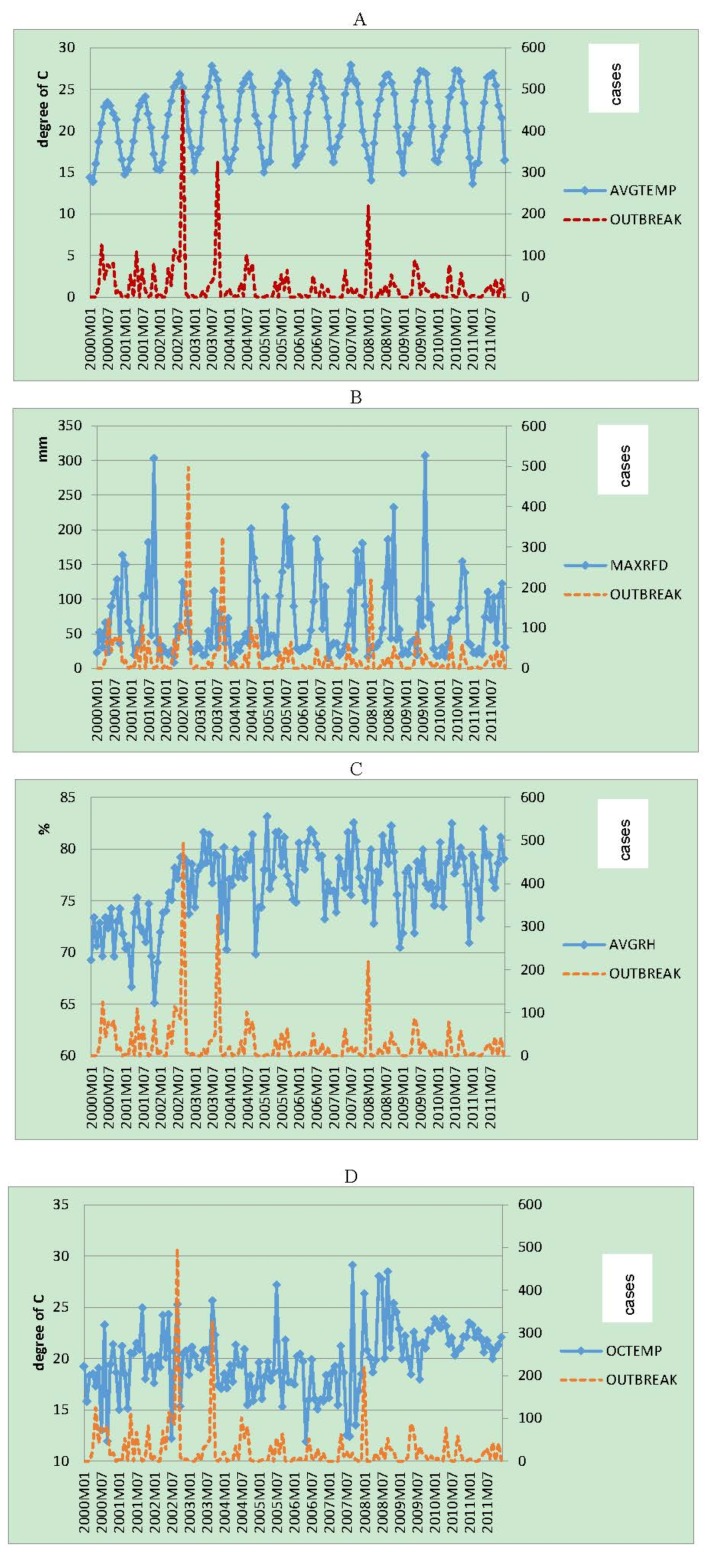

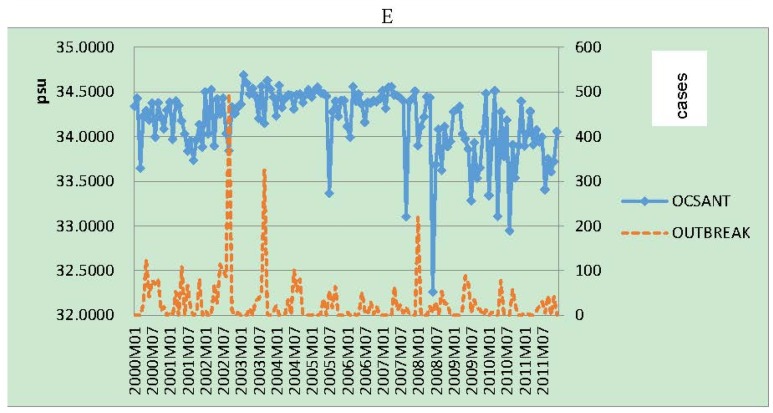
Dashed red line: Monthly incidence of *Vibrio parahaemolyticus* outbreaks in Taiwan from January 2000 to December 2011 compared to climatic variables for the same period: (**A**) average temperature (AVGTEMP); (**B**) Maximum daily rainfall (MAXRFD); (**C**) average relative humidity (AVGRH); (**D**) average ocean temperature (OCTEMP); (**E**) average ocean salinity (OCSANT).

Monthly incidence of *Vibrio parahaemolyticus* outbreaks in Taiwan from January 2000 to December 2011 compared to climatic variables for the same period is shown in Figure 1. The plot of the observed *Vibrio parahaemolyticus* incidence showed three major outbreaks in Taiwan (2002/4–2002/9, 2003/6–2003/9, 2008/1). The bivariate analysis between crude climatic variables and *V. parahaemolyticus* incidence shows that the three major outbreaks were correlated to an increase of average temperature (Figure 1A), maximum daily rain fall (Figure 1B), relative humidity (Figure 1C), and a slightly increase of ocean temperature (Figure 1D). *V. parahaemolyticus* incidence was not clearly correlated to monthly average ocean salinity (Figure 1E). An annual seasonality was identified for all these climatic variables.

As a first step in ARIMA modelling, we made the response series stationary, *i.e*., the monthly *V. parahaemolyticus* outbreaks count in Taiwan. To test of stationarity, the Augmented Dickey-Fuller (ADF) unit root test was performed. The ADF test determined whether the autoregressive term had a unit root. The model, with the time trend specification, was:

(2) $$\Delta y_{t}=a_{0}+a_{2}t+\gamma Y_{t-1}+\sum_{i=2}^{P}\beta_{i}\Delta y_{t-i+1}+\varepsilon_{t}$$

where, *a*_0_ was a constant, *a*_2_, r the coefficients on a time trend (*t*) and lag 1 period of *y_t_*, *P* the lag order of the autoregressive (AR) process, and *β_i_* the coefficient of the lag order *p* of AR terms. The hypotheses of ADF test was Ho: γ = 0, H: *r* < 0, and MacKinnon [31] one-sided *p*-values was performed.

The testing results were shown in Table 4. The ADF test statistics were −9.20254, −10.8712 and −11.18945 for the models of zero mean without trend, single mean without trend and single mean with trend specification, respectively. It indicated that all of the *p*-values were small enough (*p* < 0.05) to cause to reject the null hypothesis that the series had a unit root, so that the time series of *V. parahaemolyticus* outbreaks was stationary.

**Table 1:** Top five pathogens and foods in food poisoning cases from 2000 to 2011 in Taiwan ^a^.

		2000	2001	2002	2003	2004	2005	2006	2007	2008	2009	2010	2011	Total
Top 5	Pathogens													
1	*Vibrio parahaemolyticus*	84	52	86	82	64	62	58	38	52	61	60	52	751
2	*Staphylococcus*	22	9	18	7	8	12	18	23	14	30	41	27	229
3	*Bacillus cereus*	5	8	4	11	7	9	10	7	12	11	46	36	166
4	*Salmonella*	9	9	6	11	8	7	8	11	14	22	27	11	143
5	Pathogenic *Escherichia coli*	1	0	0	0	0	0	2	1	1	10	11	16	42
Top 5	Food													
1	Seafood	8	5	15	8	6	7	7	4	10	4	12	23	109
2	Meat	2	0	3	4	0	2	4	6	2	3	5	2	33
3	Cereal	2	2	2	0	0	5	7	5	2	2	1	4	32
4	Vegetable	1	2	1	1	8	2	2	1	0	0	5	7	30
5	Bakery and confectionary	3	3	0	0	2	0	1	0	2	4	4	1	20
	Total of food poisoning outbreaks ^b^	208	178	262	251	274	247	265	248	272	351	503	426	3485

^a^ Adapted from food-borne disease data (in Chinese) published by Taiwan Food and Drug Administration [2]; ^b^ Total of food poisoning outbreaks covered all pathogens, natural toxins and chemicals.

**Table 2:** Pearson correlation coefficients between variables, *N* = 144.

Variable	Avgtemp ^a^	Maxtemp	Mintemp	Avgrf	Maxrfd	Avgrh	Maxrh	Minrh	Octemp	Ocsant
avgtemp	1.0000									
maxtemp	0.9971 *** ^b^	1.0000								
mintemp	0.9978 ***	0.9901 ***	1.0000							
avgrf	0.5261 ***	0.4889 ***	0.5516 ***	1.0000						
maxrfd	0.5577 ***	0.5252 ***	0.5791 ***	0.9410 ***	1.0000					
avgrh	0.4417 ***	0.4320 ***	0.4487 ***	0.4498 ***	0.3371 ***	1.0000				
maxrh	0.2354 ***	0.2590 ***	0.2127 **	0.1253	0.0678	0.7315 ***	1.0000			
minrh	0.5781 ***	0.5517 ***	0.6023 ***	0.5403 ***	0.4856 ***	0.7423 ***	0.3601 ***	1.0000		
octemp	0.0956	0.1003	0.0913	0.0875	0.1070	0.1498 *	0.1438 *	0.0963	1.0000	
ocsant	−0.2152 ***	−0.2096 **	−0.2167 ***	−0.1933 **	−0.2136 **	−0.1204	−0.0384	−0.1721	−0.4668 ***	1.0000
outbreak	0.2675 ***	0.2674 ***	0.2700 ***	0.1174	0.1402 *	0.0639	−0.0377	0.1273	0.1938 **	−0.0064

^a^ Average temperature (avgtemp), maximum temperature (maxtemp), minimum temperature (mintemp), average rainfall (avgrf), maximum daily rain fall (maxrfd), average maximum relative humidity (maxrh), minimum relative humidity (minrh), ocean temperature (octemp), and ocean salinity (ocsant); ^b^ *, ** and *** indicate significance at the 10%, 5%, and 1% level of probability, respectively.

**Table 3:** The average, maximum and minimum values of outbreak variable and climatic variables by month from 2000–2011.

Variable (Unit)		January	February	March	April	May	June	July	August	September	October	November	December	Average
outbreaks (people)	avg	22.2	1.3	5.3	10.5	49.6	29.4	44.7	28.5	105.6	7.8	15.7	2.2	26.9
	max	219	7	53	70	125	114	101	86	497	26	82	12	76.9
	min	0	0	0	0	0	0	0	0	0	0	0	0	10.4
avgtemp (°C)	avg	15.4	16.5	17.9	20.9	23.6	25.2	26.4	26.3	25.2	22.9	20.3	17	21.4
	max	16.7	19.4	19.4	22.2	24.8	26.4	27.9	27.2	26.9	24.4	21.6	18.3	22.2
	min	13.6	13.9	16.1	18.7	20.9	22.9	23.4	23	22.1	20.4	17.2	15.4	19.3
maxrfd (mm)	avg	27.7	28.7	29.2	37.7	62.9	102.4	121.4	119.9	127.2	83.1	56.9	35.8	69.4
	max	54.6	52.4	46.9	66.2	104.9	186.4	232.9	307.3	303.4	180.7	150.2	103.3	91.4
	min	17.1	19.2	17	8.6	19.4	37.1	27	43.5	36.7	15.2	17.3	9.2	46.8
avgrh (%)	avg	75.3	77	75.4	76.9	77.3	79.1	77.2	78.3	78.1	75.3	75.5	73.7	76.6
	max	80.6	83.1	79.9	81.6	81.9	82.5	80.5	82.5	82.2	79.7	81.1	79	78.8
	min	69.3	70.6	66.7	72.9	69.6	72.5	72	71	69.6	69.6	65.1	69	70.9
octemp (°C)	avg	19.8	19.8	19.9	19.7	19.5	20.8	20	20.4	21.2	19.1	19.8	20	20.0
	max	26.3	24.2	23	24.2	23.1	28	27.7	28.1	29.1	22.3	25.4	24.5	23.2
	min	15	15.8	17.1	15.1	11.9	16	12.2	12.4	11.9	13.5	15.8	17	17.2
ocsant (psu)	avg	34.2	34.1	34.3	34	34.3	34.1	33.5	33.7	34	34.1	34.2	34.3	34.1
	max	34.5	34.7	34.6	34.6	34.5	34.5	34.4	34.6	34.5	34.6	34.5	34.5	34.5
	min	33.3	33.1	33.1	33.1	33.1	32.3	27.6	30.6	33.1	33.5	33.9	33.9	32.7

**Table 4:** The Augmented Dickey-Fuller Unit Root Tests for outbreaks.

Type	*t*-Statistic	*p*-Value ^a^
zero mean without trend	−9.20254	0.0000
single mean without trend	−10.8712	0.0000
single mean with trend	−11.18945	0.0000

^a^ MacKinnon [31] one-sided *p*-values.

**Figure 2:**
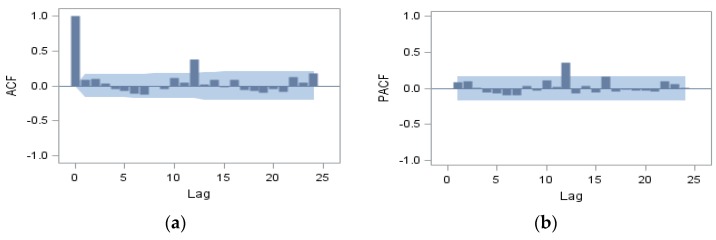
The acf and pacf of original time-series: *Vibrio parahaemolyticus* outbreaks. Gray area represents the two standard error limits; (**a**) the acf pattern; (**b**) the pacf pattern.

We further used the auto-correlation (acf) and partial auto-correlation function (pacf) plots to identify the order of the ARIMA model for the stationary series. The acf and pacf pattern of the original time-series determined the initial autoregressive (AR) and moving average (MA) order. Figure 2 showed both acf and pacf peaked at lag 12, and all other values fell between the upper and lower bounds of 2 standard errors. This pattern suggested the existence of an annual seasonal component of the original outbreak-series in the time series model.

**Figure 3:**
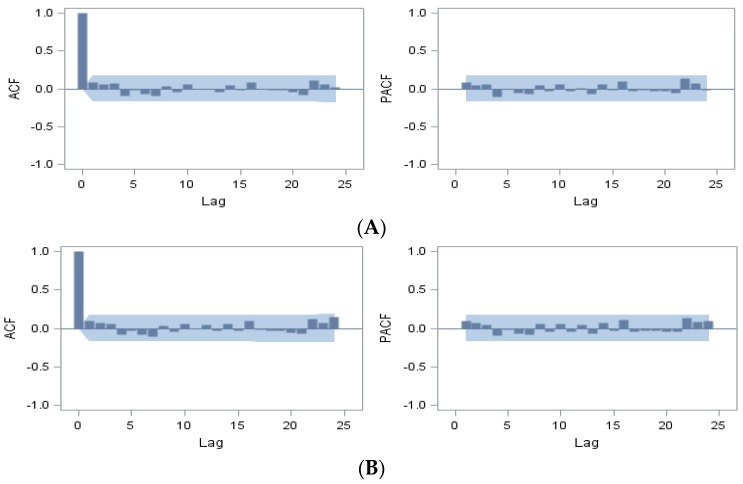

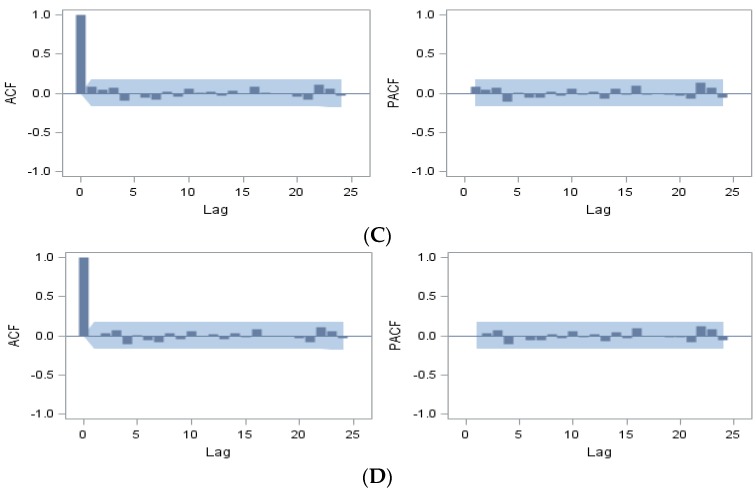
The acf and pacf pattern (3(**A**); 3(**B**); 3(**C**); and 3(**D**)) for residuals of ARIMA(1,0,0)_12_, ARIMA(0,0,1)_12_, ARIMA(1,0,1)_12_, and ARIMA(1,0,0) × (1,0,1)_12_, respectively. Gray area represents the two standard error limits.

**Figure 4:**
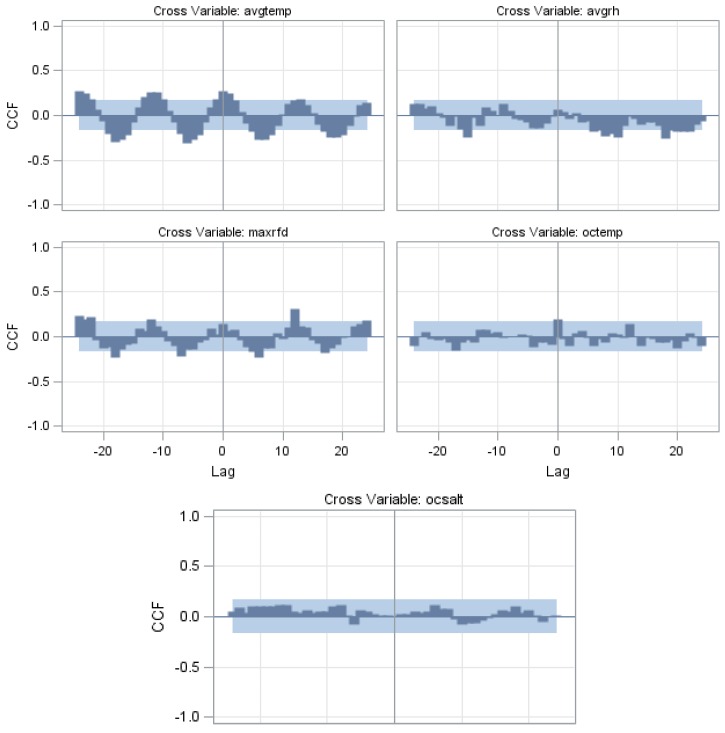
The cross-correlation coefficients of *V. parahaemolyticus* outbreaks with climatic variables: avgtemp, avgrh, maxrfd, octemp, and ocsant in Taiwan, 2000–2011. Gray area represents the two standard error limits.

To account for annual seasonality, we fitted several further univariate ARIMA models with different orders and subsequently excluded any models in which the residuals exhibited autocorrelation. Of which four univariate models, ARIMA(1,0,0)_12_, ARIMA(0,0,1)_12_, ARIMA(1,0,1)_12_ and ARIMA(1,0,0) × (1,0,0)_12_ (null model#1, model#2, model#3, and model#4) had showed better predictive Root Mean Square Error (RMSE) and lowest Akaike Information Criterion (AIC). The estimated models were tested for residuals autocorrelation. The presence of autocorrelation was investigated using the acf, the pacf and the correlogram reported in the Figure 3A–D. Figure 3 indicated all of the autocorrelation coefficients of the residuals did not differ significantly from zero with all acf and pacf values fell between the upper and lower bounds of 2 standard errors. All the performed diagnostic statistics indicated that the models pass all the tests. Their maximum likelihood estimation (MLE) estimated coefficients were summarized in Table 5. Of these four univariate models, ARIMA (1,0,1)_12_ had the best predictive RMSE and lowest AIC. We, therefore, used the model ARIMA (1,0,1)_12_, with AR at lag 12 and MA at lag 12, as the baseline univariate model for further comparisons. The selected model could be written as follows:

(3) $$\left(1-0.7526B^{12}\right)y_{t}=8.4141+\left(1-0.6836B^{12}\right)\varepsilon_{t}$$

where, *y_t_* represented the *V. parahaemolyticus* outbreaks. In order to incorporate the climate variables as input variables to the baseline univariate model, we first used ccf to examine the correlations between *V. parahaemolyticus* outbreaks and the environmental series. Figure 4 indicated that there were significant correlations (based on the two standard error limits) of outbreaks with monthly average temperatures at lag 0 to 8, monthly average relative humidity at lag 8 to 10, monthly maximum daily rainfalls at lag 7 and ocean temperature at lag 0. It was therefore evident that the original time-series was correlated with these climatic factors. Subsequently, the baseline univariate model (ARIMA (1,0,1)_12_) with one or more climatic variables included were estimated. Several models were computed and only models with statistically significant coefficient were selected, ensuring the non-autocorrelation of residuals at 5% significant level. The estimated coefficients of ARIMA(1,0,1)_12_ for *V. parahaemolyticus* outbreak series with climatic variables were shown in Table 6. For these covariate models, the best fit was obtained from ARIMA(1,0,1)_12_ with average temperature, current and one month previous maximum daily rainfall, current and 9 months previous relative humidity, ocean temperature, and ocean salinity of 6 months previous as covariates had the best prediction RMSE and lowest AIC. The selected model could be written as follows:

*y_t_* = −310.1019 − 0.0211 *y_t−12_* + 3.5590 avgtemp_t_ − 0.0151 maxrfd_t_ − 0.1734 maxrfd_t-1_ + 0.9559 avgrh_t_ − 4.5770 avgrh_t-9_ + 4.5140 octemp_t_ +13.4580 ocsant_t-6_ + 0.5153 *ε_t−12_* + *ε_t_* (4)

where, *y_t_* represented the *V. parahaemolyticus* outbreaks, *y_t−_*_12_ 12 months previous outbreak, avgtemp_t_ current average temperature, maxrfd_t_ current maximum daily rain fall, maxrfd_t-1_ one month previous maximum daily rain fall, avgrh_t_ current average relative humidity, avgrh_t-9_ 9 months previous average relative humidity, octemp_t_ current ocean temperature, and ocsant_t-6_ ocean salinity of 6 months previous. Comparing the model with the baseline univariate model (ARIMA(1,0,1)_12_), we found that including the environmental input series improved the AIC by 1.57% and the predictive RMSE by 2.54% compared to the baseline model.

**Table 5:** The estimation results of ARIMA model for *Vibrio parahaemolyticus* outbreak series: univariate models ^b^.

Variable	ARIMA(1,0,0)_12_^a^	ARIMA(0,0,1)_12_	ARIMA(1,0,1)_12_	ARIMA(1,0,0) × (1,0,1)_12_
Intercept	24.5430 *** ^b^	26.4550 ***	8.4141 ***	14.9992
AR at lag1				0.0636
AR at lag 12	0.3826 ***		0.7526 ***	0.7995 ***
AR at lag 13				−0.0473
MA at lag 12		0.3790 ***	−0.6836	−0.6372 ***
AIC	10.8494	10.8170	10.7066	10.7647
RMSE	54.0868	53.2847	49.7958	50.3811

^a^ The four univariate models can be represented as follows: model#1, ARIMA(1,0,0)_12_ : (1 − *Φ_1_B*^12^)*y_t_* = *u* + *ε_t_*, model #2, ARIMA(0,0,1)_12_ : *y_t_ = u* + (1 + *ΘB*^12^) *ε_t_*, model #3, ARIMA(1,0,1)_12_ : (1 − *Φ_1_B*^12^)*y_t_* = *u* + (1 + *ΘB*^12^)*ε_t_*, model #4, ARIMA(1,0,0) × (1,0,1)_12_: (1 − *Φ_1_B*^12^)(1 − *ϕB*)*y_t_* = u + (1 + *ΘB*^12^)*ε_t_*. where, *y_t_* is outbreak; *ε_t_* is a white noise process; *u* is intercept; *Φ*, *Θ* and *ϕ* are estimate parameters, and *B* is lag (or back) operator; ^b^ *** indicate significance at the 1% level of probability, respectively.

The best fit model indicated that average temperature, ocean salinity of 6 months previous, and ocean temperature were all significantly and positively related to *V. parahaemolyticus* outbreaks. Thus outbreaks might increase when increasing in current temperature or ocean temperature or, when increasing in ocean salinity of 6 months previous. However, there were significant negative relationships of outbreaks with maximum daily rainfall of a month previous or relative humidity of 9 months previous. This relationship meant that increased heavy rain or increased relative humidity 9 months previously may reduce current outbreaks.

**Table 6:** The estimation results of ARIMA(1,0,1)_12_ for *Vibrio parahaemolyticus* outbreak series with climatic variables: Covariate models.

Variable	Covariate #1	Covariate #2	Covariate #3	Covariate #4
Intercept	−344.9471	−368.2690	−364.3913 *	−310.1019
AR at lag 12		0.3518 *** ^a^		−0.0211
MA at lag 12			0.3230 ***	0.5153 ***
avgtemp	3.8521 **	4.7055 ***	2.3822	3.5590 ***
avgtemp at lag1			2.2843	
maxrfd	−0.1022	−0.1630 *	−0.1401 *	−0.0151
maxrfd at lag1	−0.1307	−0.1368 *	−0.1682 **	−0.1734 **
avgrh	1.1641	1.2892	1.2607	0.9559
avgrh at lag9	−4.2791 ***	−3.7936 ***	−4.0850 ***	−4.5770 ***
octemp	4.7350 ***	4.1967 ***	4.4053 ***	4.5140 ***
ocsant at lag6	13.0870 **	12.2781 **	12.8271 **	13.4580 ***
AIC	10.8543	10.8054	10.7346	10.5413
RMSE	51.8809	54.5196	51.3805	48.5625

^a^ *, ** and *** indicate significance at the 10%, 5%, and 1% level of probability, respectively.

## 4. Discussion

### 4.1. Key Findings

The results from our models were consistent with expectations and with results in the literature. The variables associated with food‑borne diseases were ambient temperature, ocean temperature, ocean salinity, and rainfall. Ambient temperature, ocean temperature, and ocean salinity were positively related to *V. parahemolyticus* outbreaks. Rainfall was negatively related to *V. parahemolyticus* outbreaks.

Theoretically, temperature was likely to influence the growth of *V. parahaemolyticus*. *V. parahaemolyticus* can grow well at below 10 °C, moreover, its generation time can be as fast as 10 min at ~37 °C [32]. Our empirical evidence showed that environmental temperature was the major factor determining the seasonality of growth and the geographical distribution of *V. parahaemolyticus* in shellfish [33,34]. The results from our models were largely consistent with expectations and results published in the literature. For example, there was a significant positive correlation between the mean temperature of the previous month and the number of salmonellosis notifications in the current month in five Australian cities. The increase in notifications was 4%–10% for every degree of temperature rise. Tam *et al.* [27] also investigated the relationship between ambient temperature and *Campylobacter enteritis* using time-series analysis to study short-term associations between temperature and number of Campylobacter reports in England and Wales. They also adjusted for long-term trend and seasonal patterns. They found a linear relationship between mean weekly temperature and reported *C. enteritis*.

In addition, our results also confirmed the importance of salinity as a climate predictor of seafood poisoning outbreaks. Compared to *V. vulnificus*, *V. parahaemolyticus* could tolerate higher salinity levels [35,36]. *V. parahamolyticus* could survive at salinity ranging from 10 to 34 ppt, with 23 ppt as its optimum salinity [28]. Although recent researches had considered impacts of climate change on salinity in costal and marine ecosystems [15,37], very little empirical evidence had been given on influences of salinity on seafood poisoning outbreaks. Recently, Young *et al.* cconducted meta-analysis to exam impacts of salinity on *V. parahaemolyticus* in oysters at harvest and in harvest waters [38]. They found no consistent relationship for water salinity. They further explained this might be due to poor reporting of study sampling methods and quantitative outcome data from collected articles. We thus encourage further research to investigate the impact of salinity in other regions or in other waterborne pathogens.

Rainfall was negatively related to *V. parahemolyticus* outbreaks as in covariate #3 and covariate #4. Deepanjali, Kumar and Karunasagar [34] also found that *V. parahaemolyticus* abundance was high during the dry season and low after rainy seasons. This might result from pathogen concentrations in estuaries being diluted by heavy rain thus reducing contamination of oysters and other filter feeding shell fish. In both Brisbane and Townsville rainfall was also a significant factor in regression models of climate effects on cases of *Salmonella* infection [26].

Furthermore, our findings also indicated that ambient temperature, ocean temperature, ocean salinity had higher impacts than rainfall on microbial seafood safety. This echoed reports in the literature showing that temperature change plays an important role in food‑borne diseases [22,23,26,27]. In distinction to other studies of food pathogen outbreaks, we also proved here the importance to *V. parahaemolyticus* outbreaks not only of ambient temperature, but also of ocean factors such as ocean temperature and ocean salinity. Marques *et al.* [11] contend that a combination of environmental and genetic factors might play a key role in the presence or absence of virulent *Vibrio* spp. because it may alter the deposition of material in bivalve shells or the composition of plankton exoskeletons. This will in turn directly change the habitat of *Vibrio* spp., so forcing the organism to adapt genetically and produce strains with great virulence. We likewise demonstrated that knowing the effects of multiple climate factors on food‑borne diseases was necessary for an intensive examination of accuracy in predicting disease. Moreover, effort should also be devoted to individualize more precisely the consequences of these interactions on *V. parahaemolyticus* infections in order to better identify control and mitigation measures [39].

Overall, increases of seafood contamination by *V. parahaemolyticus* in Taiwan due to climate changes could be explained as below. *V. parahaemolyticus* is part of the natural flora of estuarine and coastal marine environments. Climate change will likely influence the vulnerability of estuaries to eutrophication in several ways, including changes in temperature, sea level, and exchange with the coastal ocean and salinity [15,40,41]. This then will influence aquatic animals, which are vulnerable to climate change because their related metabolic processes are influenced by water temperature, salinity, and oxygen levels [42]. This in turn may favour a group of potentially emerging microbiological pathogens, the marine *Vibrios*, which are a genus of thermodependent bacteria which thrive in naturally warm sea water. Furthermore recent studies regarding climate impacts on marine systems in Taiwan has provided some evidence. For example, Chang *et al.* [43] and Lee *et al.* [44] observed that extreme weather and marine environmental changes induced by climate change could harm the marine fish population and aquaculture. Lu *et al.* [45] also observed that increased sea surface temperatures could causes fluctuations in the presence of cold-water and warm-water fishes and in the timing of fishing seasons in coastal zones of the Kuroshio Current and China Coastal Current. Chou *et al.* [21] also proved that climatic variations could influence diarrhea-associated morbidity in Taiwan. Thus, we can speculate that the increased seafood contamination by *V. parahaemolyticus* in Taiwan could be caused by climate changes.

### 4.2. Limitations

This research made an important contribution to the academic literature and provided the potential for positively influencing risk management practice; there were nonetheless limitations that provide opportunities for further research. First, our results were only for Taiwan. To generalise our findings we encouraged the implementation of similar studies in other countries. Second, we evaluated only temperature, humidity, and rainfall as predictors. We support, therefore, the inclusion in future studies of other oceanic factors, such as ocean turbidity, dissolved oxygen, and pH. For example, Parveen *et al.* [33] indicated that dissolved oxygen might increase *V. parahaemolyticus* abundance. Third, our analysis found that key predictors were related to the number of cases of *V. parahaemolyticus* infection. We suggested therefore that future studies should investigate the individual effects of these predictors on fishery production, fishery manufacturing, and the distribution system [10,46].

## 5. Conclusions

This research provided empirical evidence for the relationship between *V. parahaemolyticus* outbreaks and climatic change factors. Our results showed that average temperature, ocean salinity of 6 months previous, and ocean temperature were all significantly and positively related to *V. parahaemolyticus* outbreaks. However, there were significant negative relationships of outbreaks with maximum daily rainfall of a month previous or relative humidity of 9 months previous. Our findings also indicated that ambient temperature, ocean temperature, ocean salinity had higher impacts than rainfall on microbial seafood safety. Overall, our findings predicted that, in Taiwan, food poisoning caused by *V. parahaemolyticus* was related with climate factors. It is hoped that the findings of this research will help to guide public health and community interventions to protect Taiwanese consumers. Thus, future research into the health impacts of extreme weather events, and early warning and response tools is required.
